# Millisecond X-ray reflectometry and neural network analysis: unveiling fast processes in spin coating

**DOI:** 10.1107/S1600576724001171

**Published:** 2024-03-15

**Authors:** David Schumi-Mareček, Florian Bertram, Petr Mikulík, Devanshu Varshney, Jiří Novák, Stefan Kowarik

**Affiliations:** a Physikalische Chemie, Graz University, Heinrichstraße 28, Graz, Steiermark 8010, Austria; b Deutsche Elektronen-Synchrotron DESY, Notkestraße 85, 22607 Hamburg, Germany; c Department of Condensed Matter Physics, Faculty of Science, Masaryk University, Kotlářská 2, Brno 61137, Czechia; d Central European Institute of Technology, Purkyňova 123, Brno 621 00, Czechia; Uppsala University, Sweden; The European Extreme Light Infrastucture, Czechia

**Keywords:** X-ray reflectometry, spin coating, neural network analysis, X-ray reflectometry, millisecond XRR

## Abstract

X-ray reflectometry (XRR) is a powerful tool for probing the structural characteristics of nanoscale films and layered structures, which is an important field of nanotechnology and is often used in semiconductor and optics manufacturing. This study introduces a novel approach for conducting quantitative high-resolution millisecond monochromatic XRR measurements. This is an order of magnitude faster than in previously published work. Quick XRR (qXRR) enables real time and *in situ* monitoring of nanoscale processes such as thin film formation during spin coating. A record qXRR acquisition time of 1.4 ms is demonstrated for a static gold thin film on a silicon sample. As a second example of this novel approach, dynamic *in situ* measurements are performed during PMMA spin coating onto silicon wafers and fast fitting of XRR curves using machine learning is demonstrated. This investigation primarily focuses on the evolution of film structure and surface morphology, resolving for the first time with qXRR the initial film thinning via mass transport and also shedding light on later thinning via solvent evaporation. This innovative millisecond qXRR technique is of significance for *in situ* studies of thin film deposition. It addresses the challenge of following intrinsically fast processes, such as thin film growth of high deposition rate or spin coating. Beyond thin film growth processes, millisecond XRR has implications for resolving fast structural changes such as photostriction or diffusion processes.

## Abstract 2

This work demonstrates high-resolution X-ray reflectometry measurements with millisecond time resolution as a quantitative method for fast *in situ* measurements.

## 1. Introduction

X-ray scattering has great appeal for *in situ* investigations due to its remote probing capability, allowing measurements to be conducted in diverse environments such as under vacuum, at atmospheric pressure and in liquids (Festersen *et al.*, 2018 ▸; Pietsch *et al.*, 2004 ▸; Suryanarayana & Norton, 1998 ▸; Benediktovich *et al.*, 2014 ▸). X-ray reflectometry (XRR) is a widely used technique with exceptional accuracy and atomic-scale precision in determining the structure of interfaces and thin films (Tolan, 1999 ▸; Holý *et al.*, 1999 ▸; Braslau *et al.*, 1988 ▸; Skoda *et al.*, 2017 ▸; Russell, 1990 ▸; Kowarik *et al.*, 2006 ▸; Pietsch *et al.*, 2004 ▸; Daillant & Gibaud, 1999 ▸). However, the application of XRR to time-resolved processes is somewhat constrained. The conventional approach to XRR involves scanning both the sample and the detector, which typically necessitates several seconds or minutes for a single scan, depending on the specific setup and scan parameters employed. Additionally, the XRR measurement covers a very large intensity range of several orders of magnitude, necessitating long integration times or high-flux X-ray sources. This slow speed, however, does not suffice to study many fast processes at the nanoscale, such as thin film deposition at fast industrially relevant growth rates, diffusion or intercalation processes, for example, in batteries or light-induced sample changes in photostrictive films (Procházka *et al.*, 2017 ▸), or thin films containing molecular switches (Weber *et al.*, 2016 ▸).

Although many variations of XRR, referred to broadly as quick X-ray reflectometry (qXRR) (Sakurai *et al.*, 2007 ▸; Ogawa *et al.*, 2013 ▸), have been developed to overcome this limited time resolution, they generally place severe constraints on the source and/or sample, which make them incompatible with certain applications (Joress, Brock *et al.*, 2018 ▸; Joress, Arlington *et al.*, 2018 ▸). In recent work, *in situ* material growth using qXRR at a measurement speed of 100 ms per curve was performed with a combination of an angular-dispersive monochromatic X-ray beam and a fast 2D detector (Joress, Brock *et al.*, 2018 ▸). With a reduction of the diffuse scatter by rotating the sample such that the surface normal is out of the plane of the incident beam (Voegeli *et al.*, 2017 ▸), the velocity of the qXRR increased up to 10 ms per curve when a high-brightness source was used (Joress, Arlington *et al.*, 2018 ▸). With a polychromatic X-ray source (Kowarik *et al.*, 2007 ▸), an XRR curve was also obtained in 10 ms (Matsushita *et al.*, 2013 ▸).

Without the use of an angular-dispersive monochromatic X-ray beam, the measurement requires more time; however, a parallel X-ray beam can be employed. The fastest measurement made with the classical angular scanning method was obtained with 10 ms per point and around 10 s of total measurement time (Mocuta *et al.*, 2018 ▸). An alternative way to increase the speed of the measurement is to adapt the geometry of the sample. A dynamically bent sample was introduced with an *in situ* measurement of rapid heating of the sample (Liu *et al.*, 2017 ▸). Another approach has been published whereby the sample had a certain roughness that allowed one to acquire reflectivity at multiple angles simultaneously; however, this severely limited the choice of substrate (Fujii *et al.*, 2020 ▸).

Other previously published work (Lippmann *et al.*, 2016 ▸) demonstrated that an experimental setup with a rotating wedge can be applied to remove the constraint of slow sample movements. By employing a static 2D detector and placing the sample onto a piezo-rotating stage where the rotation axis is not perpendicular to the X-ray beam, XRR curves can be collected quickly on the detector. This leads to an opportunity to perform qXRR measurements where the main constraint is the speed and stability of the rotating stage. Here we extend this method beyond the previously published time resolution of 2 s per reflectivity curve via usage of a high-performance detector, high flux at the PETRA III synchrotron radiation source and a fast-rotating stage.

Here we demonstrate qXRR with a remarkable time resolution of 1.4 ms per curve, while still obtaining quantitative data suitable for fitting in a wide *q* range (0.33 Å^−1^) and at high resolution (0.0016 Å^−1^). To validate the data quality of qXRR, we employ it in a two-layer sample structure of gold/silica/silicon. Further, we study millisecond XRR during spin-coating, offering a glimpse into the dynamic evolution of the spin-coating layer with millisecond precision where the rapid initial thinning of the PMMA layer was observed as well as evaporation of the solvent from the film. The combination of millisecond XRR and spin coating presents a truly unique opportunity for real time exploration of thin film growth and development – a valuable methodical asset in the quest for advancing novel materials and groundbreaking applications.

## 2. Experimental setup

**Figure 1:**
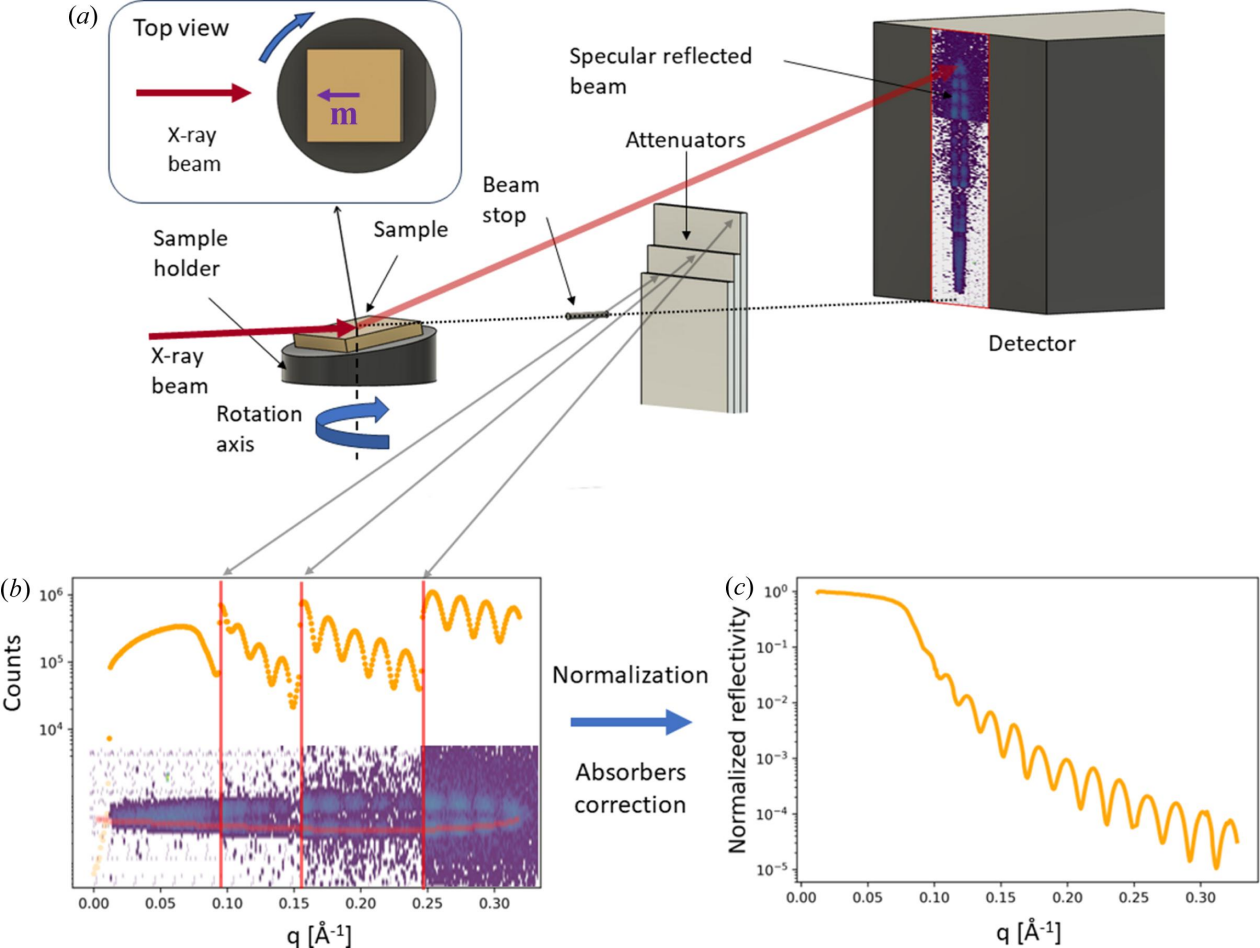
(*a*) Schematic view of the experimental setup with the beam stop and attenuator positions. (*b*)–(*c*) Scheme of the pipeline for data extraction from the detector and normalization. (*b*) One extracted branch (one qXRR) directly from the detector signal with visible attenuator edges and an area covered with the beamstop. (*c*) Final qXRR curve after the application of the absorption correction, footprint correction and normalization.

To enable qXRR measurements we used the high-flux and high-resolution P08 beamline at DESY, PETRA III (Seeck *et al.*, 2012 ▸), where the energy of the primary beam was set at 18.00 keV. We utilized a compact spin coater, custom-built in-house using 3D FDM printing, with its rotational axis perpendicular to the X-ray beam axis. Atop the spin coater, we affixed a sample holder with a wedge-shaped design [Fig. 1 ▸(*a*)]. The sample holder is removable, and for our experiments, we employed holders featuring wedge angles (ω_max_) of 1 and 2°. Consequently, as the spin coater rotated, the sample altered its inclination to the beam, ranging from 0° to either 1 or 2°, depending on the specific holder used.

To establish a reference point, we designated the minimum spin coater rotation angle denoted φ = 0° when the sample reached its lowest inclination to the beam (ω = 0°) [Fig. S1(*b*), (i)]. Subsequently, we can define that, for φ = 90° [Figs. S1(*c*), (iii) and Fig. 1 ▸], the inclination of the sample to the beam is ω = ω_max_ and, for φ = 180°, we have again ω = 0° and no reflection. During the rotation from φ = 0° to φ = 90°, one XRR curve can be collected, and a second can be collected during the rotation from φ = 90° to φ = 180° (Fig. S2). For a range φ = (180, 360°) the sample with the holder blocks the beam as the high side (back side) of the wedge is hit. Note that our setup is slightly different from that of Lippmann *et al.* (2016 ▸), where one XRR curve is acquired not in a quarter turn but in a half turn. This has the advantage of continuous scanning, instead of the intermittent nature of our experiment with two curves in half a turn and no signal for another half turn. However, our setup increases the speed per qXRR scan by a factor of two. The rotating-sample qXRR stands out from other qXRR methods in the literature (Joress, Brock *et al.*, 2018 ▸; Joress, Arlington *et al.*, 2018 ▸) as it is easier to implement an additional motor at existing beamlines instead of modifying the X-ray source by creating a fan-shaped X-ray beam with a polycapillary. It is also crucial to underscore that our approach only necessitates straightforward geometrical corrections to obtain a normalized XRR curve, and no experimental normalization via measurement of an angular intensity profile (Joress, Brock *et al.*, 2018 ▸; Joress, Arlington *et al.*, 2018 ▸) is needed, which may introduce errors, *e.g.* at the important total reflection edge.

We employed the Eiger2 X 1M detector which gives a good *q* resolution due to its compact pixel size. To reduce the count rate in the experiment to a value within the dynamic range of the detector (10^7^ photons s^−1^ pixel^−1^), three motorized absorber plates were placed after the sample in front of the detector, as illustrated in Fig. 1 ▸(*a*). A beam stop was employed to protect the detector from the direct beam and reduce air scattering. Note that the residence dwell time of the beam in our qXRR approach can be as low as 1 µs, effectively limiting the maximal count rate to 10 counts per pixel in each curve to avoid photon bunching within the single photon counting time interval. Although this is easily accomplished via absorbers, it severely limits the count statistics which are essential for qXRR, and the use of integrating detectors instead of single photon counting would be advantageous for future experiments. The curve the reflected beam follows on the detector and the extraction of the specularly reflected beam during the collection of the two XRR curves are discussed in the supporting information.

For the rotation stage, we employed a motor from a 3.5′′ hard disk, as it can reach high rotation speeds of up to 10 500 rpm and the actual speed can be controlled via a brushless motor controller. Importantly, it has low wobble and also little height change between standstill and rotating states so that the movement is precise and alignment can also be performed on a motor at a standstill. To synchronize the rotation with the detection process, the sample holder was equipped with a light barrier that triggered at a specific φ once per rotation. This light barrier triggered the detector system after a user-set sleep duration, which allowed us to set the exact start of collection to the point when φ reached 0°. A second time constant determined the collection time of the detector so that it acquired data for the full duration of two XRR curves. These time constants were meticulously calculated based on the rotation frequency and verified before the actual measurement by analyzing the shape of the XRR signal observed on the detector.

## 3. Methods

### 3.1. Data extraction and normalization

To extract the reflectivity curve from the detector image, we developed a Python-based pipeline that incorporates all necessary corrections [Figs. 1 ▸(*b*) and 1 ▸(*c*)], namely footprint correction, absorber correction and residence dwell time normalization along the curved shape of the beam on the detector. While φ alters linearly for the sample rotating at a constant speed, the angle of incidence is not changing linearly. Therefore, the collected X-ray signal needs to be normalized to correct for the different exposure times for different pixels corresponding to different ω. The Python pipeline is available at the GitHub repository https://github.com/DavidMarecek2/Millisecond-XRR and background can be accessed in the supporting information of this article. Utilizing equation (S8) from the supporting information, we established a relation between the illuminated pixels on the detector and the corresponding sample tilt (ω).

In our analysis, we aimed to extract both branches (described in the supporting information) of the XRR data independently whenever possible. However, there were instances where distinguishing between the branches was not feasible. In such cases, we combined the signals and divided the counts by two. This scenario typically occurred near the total reflection edge and is close to the direct beam, where both branches illuminated nearby pixels (as depicted in the supporting information). In regions where we could differentiate between the branches, we counted all the counts from the pixels corresponding to the width of the primary beam. Subsequently, we reconstructed the XRR curve by taking into account the detector absorbers. Afterwards, we applied the footprint correction as described in the supporting information, to obtain a corrected XRR curve. Note that all these corrections are of a simple geometrical nature and can be precisely calculated.

### 3.2. Spin-coating setup

With our qXRR setup, we were able to observe the spin-coating of polymethyl methacrylate (PMMA). Preparation of the PMMA solution was performed according to a previously reported procedure (Le *et al.*, 2012 ▸). PMMA (∼350 kDa) was dissolved in toluene with concentrations of 2.5 or 5 g l^−1^, and the solution was heated to 45°C until no solid particles were visible. It was sonicated for 15 min using an ultrasonic bath and filtered through a 0.2 µm pore size polytetrafluoro­ethylene filter to remove any remaining aggregates and dust particles.

To facilitate the real time PMMA spin-coating qXRR measurement, we set up a dedicated chamber containing the spin coater. The chamber was equipped with X-ray-transparent Kapton windows (changed after each spin-coating process) to allow the X-rays to enter. We flushed the chamber with nitro­gen to minimize potential beam damage of the layer from the X-ray-induced ozone. At the top of the chamber, we installed an electromechanically controlled pipette loaded with a PMMA solution suspended in toluene. For the substrates, we utilized commercial silicon wafers (NanoLane) with a thermally grown layer of SiO_2_ measuring 100 nm in thickness. The wafers were used as received, after the removal of the protective film. The silicon wafer was carefully positioned on the spin coater, and the PMMA solution was then dropped onto the rotating sample using the remotely triggered pipette mechanism. We measured the development of the thin film with qXRR for the next 3 min. Our measurements encompassed a range of rotation speeds, from 30 to 175 Hz as shown in Table 2. Additionally, two different PMMA concentrations, 2.5 and 5 g l^−1^, allowed us to investigate the impact of rotation speed and PMMA concentration on the resulting qXRR measurements.

### 3.3. Data fitting

For the analysis of data obtained from the spin-coating experiments, we employed two curve-fitting analysis tools: *refnx* (Nelson & Prescott, 2019 ▸) curve-fitting analysis based on the advanced genetic algorithm and *mlreflect* (Greco *et al.*, 2022 ▸) based on a neural network (NN) algorithm. *mlreflect* was employed for analyzing the large datasets of 10 000 curves generated during the spin-coating procedure as the NN analysis only takes ∼20 ms per curve fit, whereas *refnx* takes ∼2 s per curve fit.

Using *refnx* enabled us to conduct single curve fits and explore multi-layer models. We fitted a scattering length density (SLD) of 19.83 × 10^−6^ Å^−2^ for Si and 18.709 × 10^−6^ Å^−2^ for SiO_2_ on the bare sample and fixed the substrate parameters for subsequent fits of the PMMA layers. The fitting procedure involved determining the roughness of Si as 1.1 and 2.6 Å, while the thickness of SiO_2_ was fitted as 992 Å to match the fast low-amplitude XRR fringes. With these fixed parameters, we employed a one-box model for the spin-coating structure evolution, focusing solely on investigating the SLD, roughness and thickness of the PMMA layer. Using this model we subsequently simulated a one-box model NN training dataset. The trained NN model was then employed for analyzing all spin-coating runs. By combining the strengths of *refnx* and *mlreflect*, we were able to effectively analyze the spin-coating data, achieving accurate fits and harnessing the power of NN algorithms for handling the large dataset of more than 10^5^ XRR curves. For the spin-coated films at higher speeds, the data start to be noisy due to the short exposure time. Here the NN approach performs well (Mareček *et al.*, 2022 ▸) because of the high NN noise tolerance. This comprehensive approach allowed us to gain valuable insights into the thin film growth process and extract meaningful information from the reflectivity curves.

## 4. Results and discussion

### 4.1. Gold thin film on silicon

**Figure 2:**
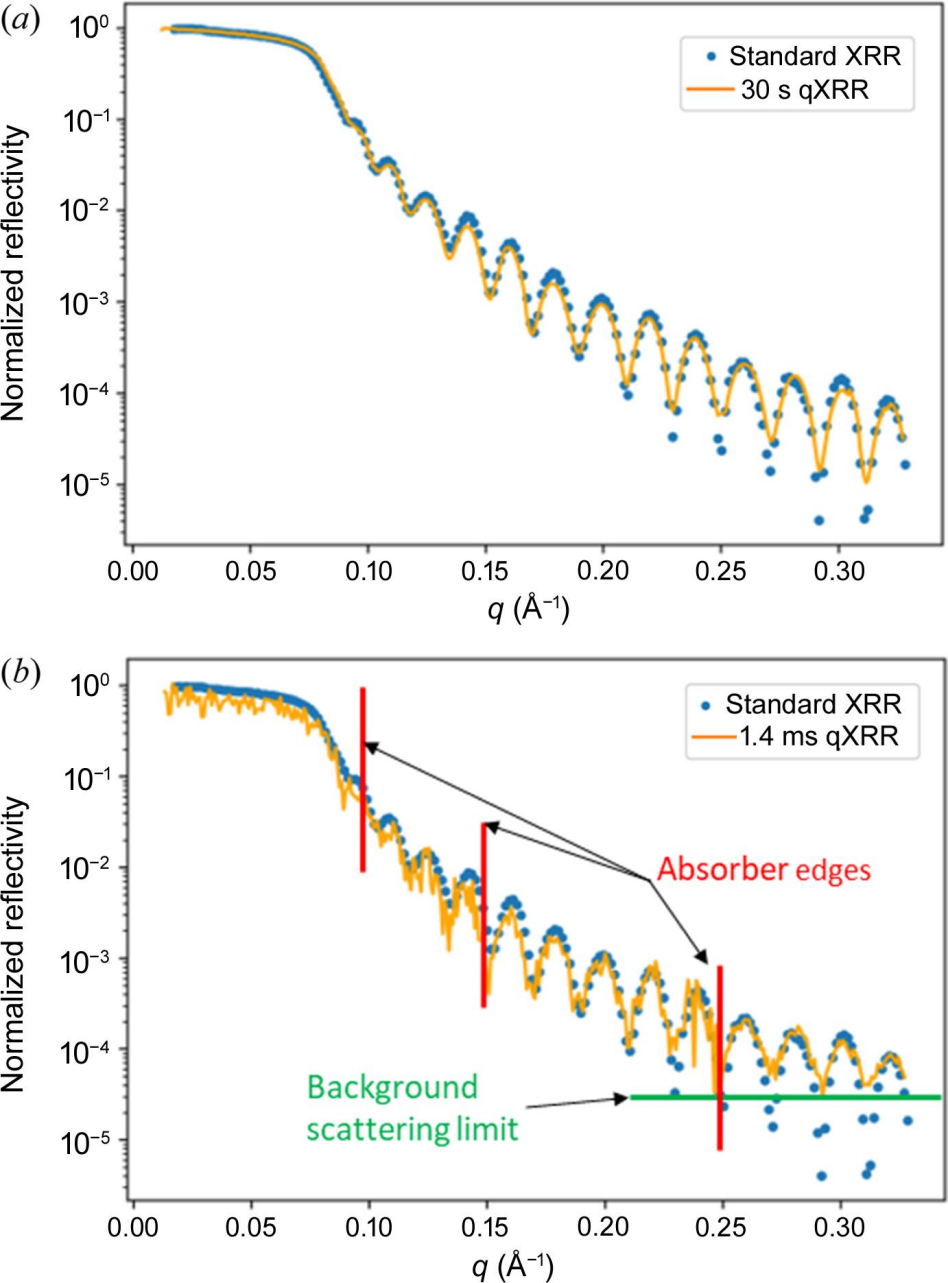
Comparison of standard XRR with our qXRR. (*a*) Long exposure time (30 s) qXRR (orange line) and the reference standard XRR (blue dots). (*b*) The measurement was performed at a rotation frequency of 175 Hz, and the resulting qXRR curve was obtained in 1.4 ms; the qXRR (orange) curve is compared with the classical XRR curve (blue dots) also shown in (*a*). In the curve noise increases up to the three absorber edges (red vertical lines) and for higher *q* background scattering limits the measurement (green horizontal line).

We used a gold-coated silicon wafer as a reference sample for a comparison between a conventional XRR curve and a rotation-based qXRR curve [Fig. 2 ▸(*a*)]. The classical angular scanning XRR curve utilized an exposure time of 1 s per point, resulting in a total measurement time of 200 s including motor movement times. On the other hand, the qXRR curve was acquired using a rotating sample with a rotation speed of 8 Hz and a total exposure time of 30 s (data collected during multiple revolutions). This side-by-side comparison serves as validation of the accuracy and reliability of our data-extraction pipeline. Some differences in the depth of the XRR minima are visible at large *q* values. This however is not a limitation of our qXRR resolution, Δ*q* = 0.016 Å^−1^. It is rather due to the background-scattering effect.

On the same sample, we conducted measurements at a maximum spinning frequency of 175 Hz, resulting in the acquisition of a single qXRR curve in an astonishingly short duration of just 1.4 ms [see Fig. 2 ▸(*b*)]. Although the qXRR curve exhibits a noticeable increase in noise levels, it remains in good agreement with the conventional XRR measurement. Notably, the increased noise in the qXRR curve just prior to absorber transitions is attributed to the lower counts registered at the detector (indicated by the red lines denoting absorber edges), resulting in elevated shot noise (Poisson noise). Furthermore, we observe that the minima in the qXRR curve, particularly the last four Kiessig fringes, appear to be less pronounced. This behavior can be attributed to reaching the photon flux limit of PETRA III. The primary beam intensity of 2 × 10^12^, the low sample reflectivity of 10^−5^ and our brief integration time of 2 µs per data point yield count rates of only a few photons per pixel. This results in the reflectivity signal reaching the background scattering limit, which in turn causes the minima to appear shallower in comparison with the reference curve.

To establish the validity of qXRR, we compared data analysis results from standard 200 s XRR measurements, 1.4 ms qXRR measurements and integrated 30 s-long qXRR measurements, using an identical model and the *refnx* fit software (Nelson & Prescott, 2019 ▸). The fitting results exhibit only minor variations, demonstrating the usefulness of qXRR. The fitting results are shown in Table 1 ▸ and the variation in the results is within the fit error range.

Our experimental setup increases the XRR speed by almost an order of magnitude compared with previous studies by going to the limits of mechanical sample movement, detector maximum count rates and synchrotron photon flux. One constraint of our qXRR setup is the rotational speed of the motor. The motor had a maximum rotation speed of 175 Hz (10 800 rpm), but faster motors exist.

Our qXRR method is also restricted by current state-of-the-art detector technology. The single photon counting rate limitations of 10 MHz (10^7^ photons s^−1^ pixel^−1^) encountered with the Eiger2 X 1M detector severely constrain the measurement. To roughly estimate this limitation, we assume that within 1 ms the X-ray beam passes 1000 Eiger pixels at constant speed, so that the beam only stays on each pixel for 1 µs. Within 1 µs, only a maximum count rate of 10 photons is possible before photon pile-up effects and saturation occur. Instead of a single photon counting detector, an integrating detector for synchrotron sources [similar to XFEL detectors such as AGIPD (Shi *et al.*, 2010 ▸)] could provide a higher dynamic range than the maximum of 5000 counts per second per pixel in our setup and thereby make it possible to use less attenuation for smoother XRR curves at low *q*.

Also, higher photon flux would benefit our measurement, as even at the current rotation speed of 175 Hz we collect only a few photons for higher *q* values [see Fig. 2 ▸(*b*)]. This challenge of measuring a wide *q* range would become even more pronounced for faster motors such as 180 000 rpm CNC high-speed spindles.

### 4.2. *In situ* qXRR spin coating

As a second application of qXRR, we observed the real time evolution of thin film growth and dynamics for PMMA spin coating. Two PMMA solutions were studied, as shown in Table 2 ▸ and described in Section 3.2. In these experiments, we were able to measure two qXRR curves per revolution of the sample which is inherently rotating during spin coating.

#### 4.2.1. Spin coating at 30 Hz

**Figure 3:**
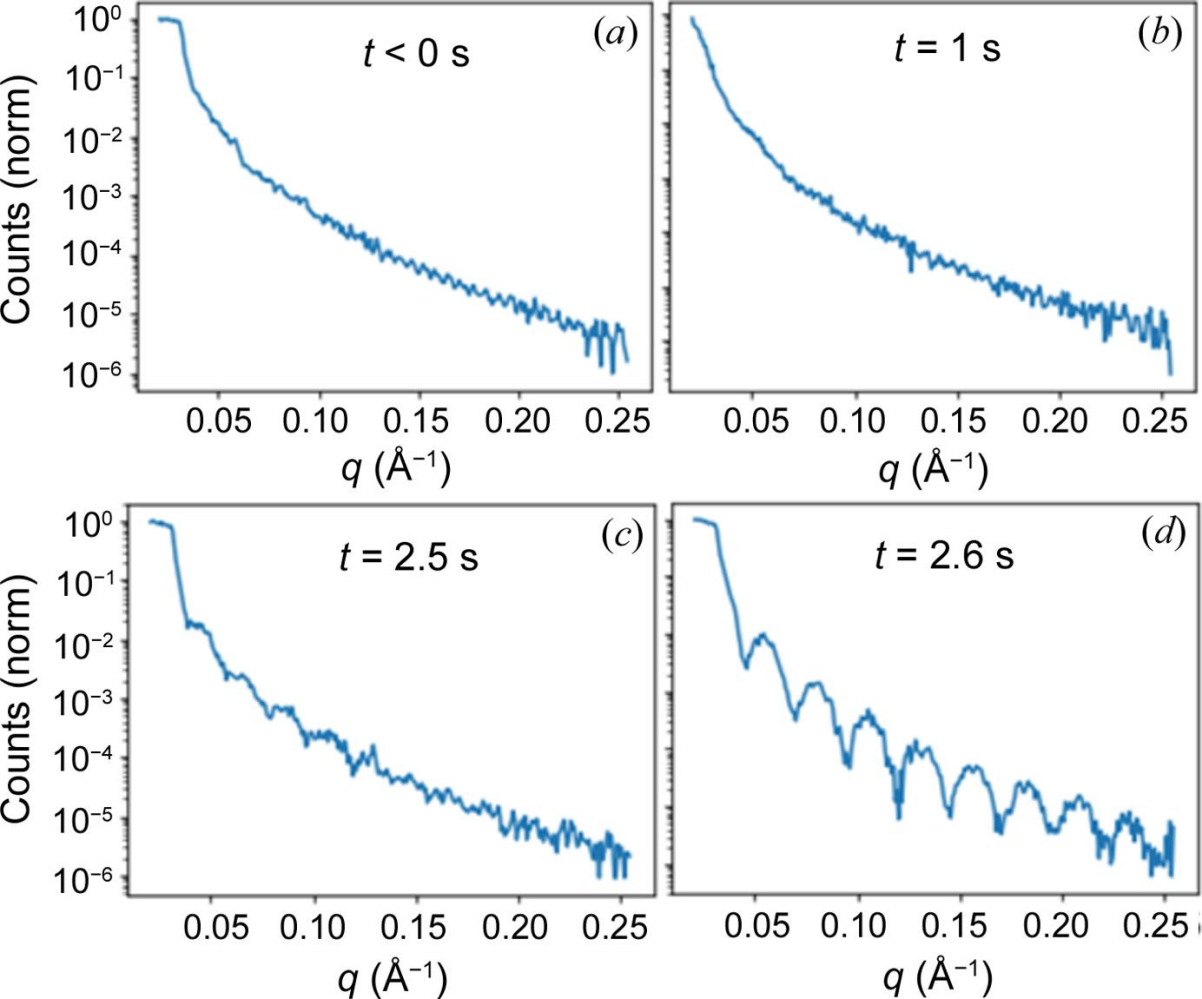
Evolution of the qXRR surveys during spin coating of the PMMA solution (5 g l^−1^) at a rotation speed of 30.3 Hz. The PMMA solution was applied to the sample at *t* = 0 ms. (*a*) The negative time corresponds to the qXRR from the clean wafer. (*b*) From 0 to 2.5 s, the beam is scattered due to droplets of PMMA solution on the substrate. (*c*) After 2.5 s, the first XRR curve with Kiessig oscillations becomes visible as the PMMA layer starts to form and exhibits an increasing level of smoothness represented by the drop of roughness. (*d*) As the layer continues to thin and become smoother, the Kiessig oscillations in the XRR curve become more pronounced.

Here we show an analysis of a toluene solution of PMMA at a concentration of 5 g l^−1^ spin coated at a rotation speed of 30.3 Hz. We use this experiment as an example of our analysis pipeline but all experiments were analyzed identically. In the beginning, we focus on the development of a thin film layer over a short time scale, as illustrated in Fig. 3 ▸. The time resolution is given by the rotation speed. Therefore we obtained two qXRR curves every 33 ms, and the time for the data collection of one qXRR curve was 8.25 ms, as noted in Table 2 ▸. During a half rotation we can collect two qXRR curves and the collection time for one qXRR curve is 1/4 of the rotation time. The investigated time frame encompasses the period starting from the application of the PMMA solution (time 0) until a time of 3 s, well beyond the point at which the film reaches a thickness where the viscosity of the film prevents further mechanical thinning (Meyerhofer, 1978 ▸).

Before the deposition of the solution, the reflectivity of the clean sample was measured from the rotating sample [Fig. 3 ▸(*a*)]. Here, narrow Kiessig oscillations of the silica layer are visible, demonstrating the high resolution of qXRR. As the SLDs of silicon and silica are very similar, this also demonstrates the ability of qXRR to resolve low-contrast SLD profiles. On deposition of the solution onto the sample, a noticeable decline in X-ray intensity is observed (not visible in the normalized data in Fig. 3 ▸, but obvious in the raw data), accompanied by the disappearance of the reflectivity edge due to X-ray scattering caused by the solution droplet [Fig. 3 ▸(*b*)]. Subsequently, we observe a gradual restoration of maximum intensity and a slow reappearance of the reflectivity edge as the film undergoes a transition to a more homogeneous thin layer [Fig. 3 ▸(*c*)]. The length of the transition depends on the rotation speed (discussed in Figs. S6 and S7). At approximately 2.5 s after deposition, we encounter the first qXRR spectra that can be fitted to determine the thickness of the PMMA film [Fig. 3 ▸(*d*)]. This moment signifies when the film has achieved sufficient overall flatness and local smoothness for XRR measurements.

**Figure 4:**
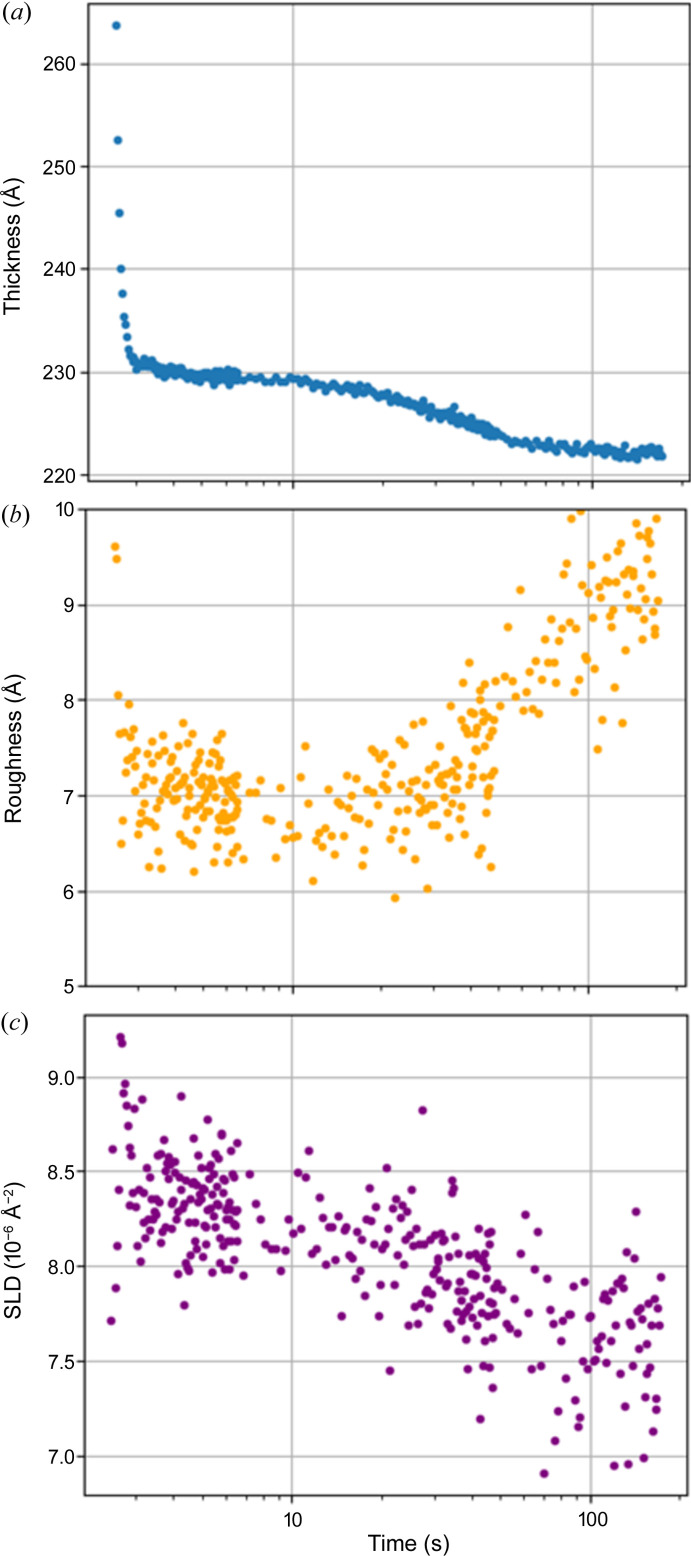
Evolution of the PMMA layer parameters during spin coating. (*a*) PMMA thickness versus time. The plot demonstrates an initial fast decay followed by a period of stable thickness, indicating equilibrium between the centrifugal force and viscosity. Subsequently, on the time scale of tens of seconds, thinning occurs due to toluene evaporation. (*b*) PMMA roughness versus time. The graph illustrates an increase in roughness attributed to toluene evaporation. (*c*) PMMA SLD versus time. The plot displays a decrease in the film SLD as toluene evaporates from the porous PMMA layer. The time interval between measurement points is 33 ms and one XRR curve was obtained in 8.25 ms.

In addition to providing information about thin film thickness, XRR measurements also allow us to determine the roughness and SLD of the film. Analyzing the time series depicted in Fig. 3 ▸, we observe that the roughness of the film is initially high, exhibiting a lack of distinct oscillations. However, once the oscillations become evident, the roughness decreases to expected values, as illustrated in Fig. 4 ▸(*b*). Notably, within this time range, we do not observe any significant changes in SLD.

On a longer time scale, the PMMA layer eventually reaches a stable state, characterized by a balance between the viscosity of the PMMA layer and the centrifugal force (Meyerhofer, 1978 ▸). At this stage, no significant changes in roughness or SLD are observed. However, approximately 10 s after the initial drop, we begin to observe a secondary thinning of the PMMA layer as the toluene evaporates from the film on the second timescale (Reisfeld *et al.*, 1991 ▸) [Fig. 4 ▸(*a*)]. The evaporation process leads to an increase in roughness [Fig. 4 ▸(*b*)] and, consequently, a decrease in SLD as the solvent disappears [Fig. 4 ▸(*c*)]. These changes are indicative of the formation of porosity within the PMMA layer, accompanied by the replacement of toluene with nitro­gen in the voids.

**Figure 5:**
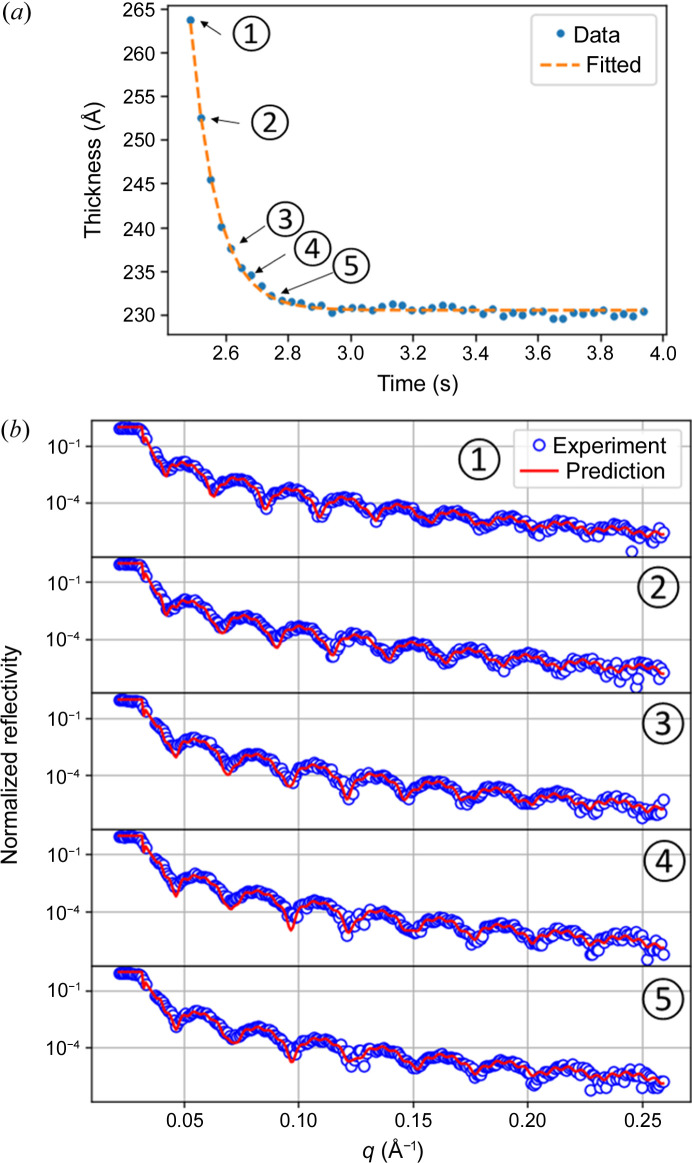
(*a*) Thickness evolution of the PMMA layer at the short time scale fitted with an exponential decay function. (*b*) Fit of the qXRR data by NN for five representative qXRR curves during the PMMA thinning (5 g l^−1^ concentration and 30.3 Hz rotation).

The measured PMMA layer thickness evolution in the early stage can be fitted with exponential decay *T*(*t*) = *T*_0_exp(−*t*/τ), where *T*(*t*) is the evolution of the thickness, *T*_0_ is the thickness at the beginning of the fit, *t* is time, and τ is a time constant depending on the solution concentration and rotation speed. Fig. 5 ▸(*a*) shows the initial thinning of the PMMA layer starting with the point when the XRR curve exhibits Kiessig oscillations. We show five XRR curves collected during the fast thinning phase and fitted with the NN in Fig. 5 ▸(*b*), where the thinning of the layer can be observed as an increasing width of the Kiessig oscillations.

#### 4.2.2. Rotation speed and concentration dependency

**Figure 6:**
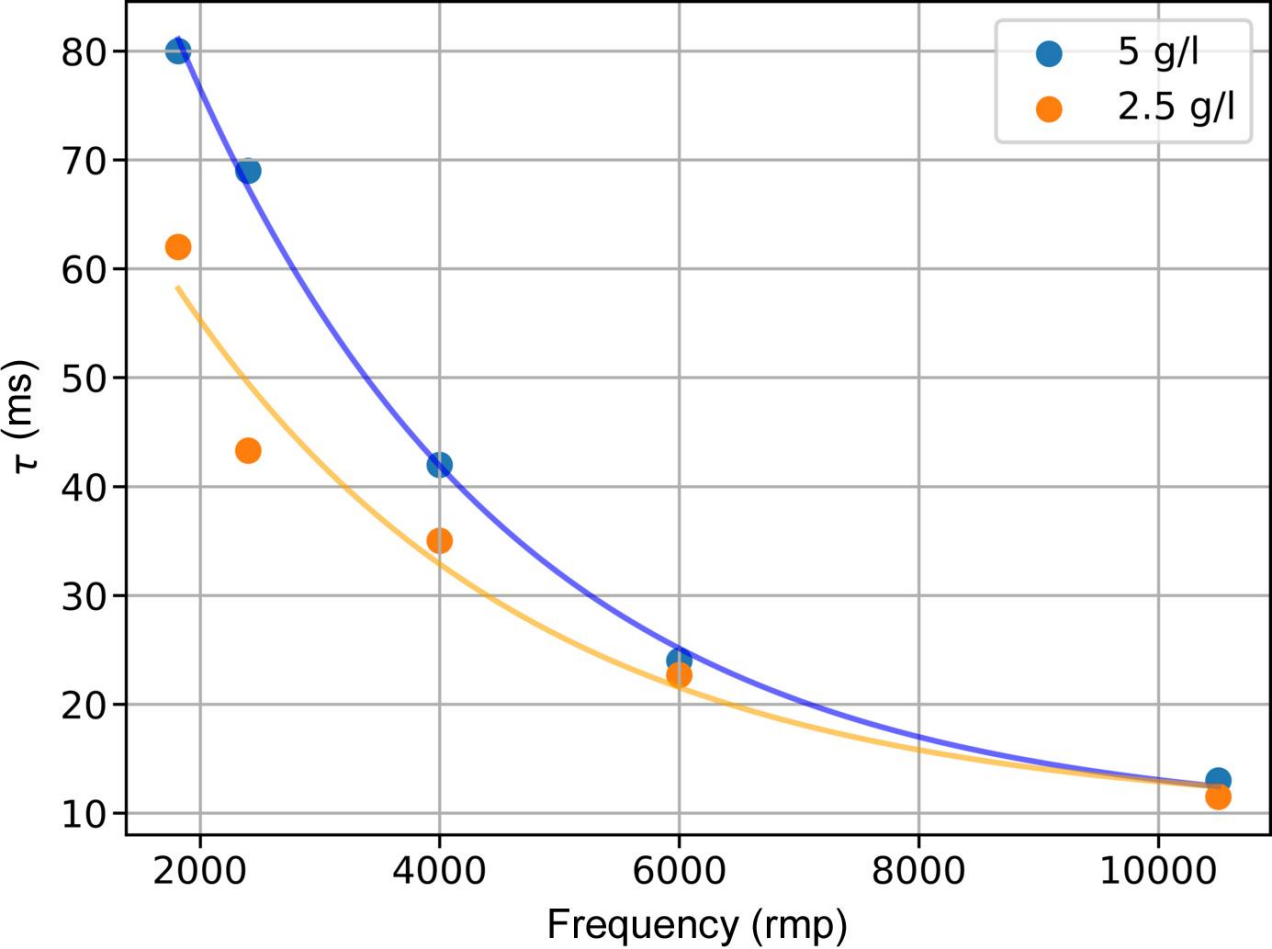
Time constant of the initial dynamic film thinning for both PMMA concentrations [2.5 g l^−1^ (orange) and 5 g l^−1^ (blue)]. The evolution is fitted with an exponential decay. The time constant τ is lower for the solution with the concentration 2.5 g l^−1^, which corresponds to the lower viscosity of the solution. For higher rotation frequencies, the time constant τ is the same for both concentrations and viscosities as the centrifugal force starts to dominate.

We investigated all the spin-coated PMMA layers shown in Table 2 ▸ in the same fashion as we investigated the first spin-coating example above. We extracted thickness, roughness and SLD from the qXRR curves. Regarding the initial thickness, we observed an exponential decay for all films, but for the faster spin-coating speeds the exponential decay was steeper, corresponding to a smaller value of τ as shown in Fig. 6 ▸ (see also Figs. S10–S18). For lower rotation speeds we can differentiate between the steepness of the exponential decay for higher (5 g l^−1^) and lower concentration (2.5 g l^−1^); however, for higher rotation speeds the centrifugal forces are dominant and the decay constant is the same for both concentrations. Moreover, the time between the application of PMMA and the observation of the first Kiessig oscillations became shorter for faster rotation speeds; this is denoted as ‘Delay’ in Table 2 ▸. This corresponds to the higher centrifugal force, which moves the solution from the sample faster and creates a flat smooth PMMA layer suitable for XRR measurement in a shorter time (Figs. S6 and S7).

On achieving mechanical stability, the PMMA film thickness stays approximately constant, with a subsequent decline attributed to the evaporation of toluene, as was already discussed above for the sample measured at 30 Hz. Notably, the duration of the stable phase and the pace of thickness reduction are dependent on the initial thickness of the PMMA layer. Thinner layers exhibit an earlier onset of thinning through toluene evaporation, leading to swifter attainment of the final thickness (Figs. S6 and S7). This observation underscores the intricate relationship between the initial conditions of the PMMA layer and the temporal dynamics of its subsequent thinning process.

Analyzing the surface structure and roughness of the film reveals consistent trends. Thicker films, prepared at lower spin-coating speeds, exhibit a decline in SLD as toluene evaporates from the PMMA layer (Reisfeld *et al.*, 1991 ▸), leaving pores that are consequently filled with nitro­gen. This coincides with an increase in roughness as the layer thins due to toluene evaporation. For the thin PMMA layer, the SLD remains consistent throughout the entire analyzed time range, indicating a higher level of smoothness and reduced porosity in comparison. The roughness remains constant initially, but towards the end of the time range, it experiences an increase due to the evaporation of a thin top layer of toluene. Nevertheless, the final roughness is still lower than that of the thick layer (Figs. S8 and S9).

Note that the one-box model, although the simplest approach, may not fully capture the complexity of the PMMA layer. The PMMA layer can be viewed as a more intricate structure, potentially exhibiting a bilayer structure (Wu *et al.*, 1994 ▸; Akers *et al.*, 2015 ▸; Van der Lee *et al.*, 2001 ▸) or even a three-layer structure (Sharma *et al.*, 2021 ▸; Bollinne *et al.*, 1999 ▸). In the bilayer approach, the spin-coated PMMA film is considered a combination of an SiO_2_–PMMA interface layer with lower density and bulk PMMA (Wu *et al.*, 1994 ▸). Alternatively, in the three-layer approach, the structure consists of an SiO_2_–PMMA interface layer, bulk PMMA and a semifluid layer on top of the sample (Sharma *et al.*, 2021 ▸).

While we were able to successfully fit our qXRR data using the simple one-box model, we also explored more sophisticated models. However, employing these complex models did not yield any significant improvement in the fit. For further investigation, we compared the performance of the two-/three-box model fits with the simple model using long exposure qXRR data, where the noise level was considerably lower. We found that all models could fit the data and the accuracy of the fits was highly comparable, with χ^2^ ≃ 3.5. This was mainly because the intermediate and semifluid layers were fitted as very thin and with SLDs close to the SLD of the bulk PMMA. The only instance where we observed a significant improvement with the more complex model was in cases involving solutions with low PMMA concentrations (2.5 and 1 g l^−1^) (Figs. S4 and S5, respectively) at low spin-coating speeds. The PMMA solution with the concentration 1 g l^−1^ was investigated only for testing purposes and only at a spin-coating speed of 30.3 Hz. Measurements of samples prepared with a more concentrated solution (5 g l^−1^) spin coated at the same speed were easily fittable with the one-box model [Fig. 5 ▸(*b*)]. The analysis is shown and discussed in the supporting information (Figs. S3–S5).

The thickness development of the PMMA layer at the short time scale has already been described in multiple publications and multiple models have been suggested (Reisfeld *et al.*, 1991 ▸; Bornside *et al.*, 1989 ▸; Higgins, 1986 ▸; Meyerhofer, 1978 ▸). The models were compared (Mouhamad *et al.*, 2014 ▸) and variations are mostly visible at the time immediately after the solution application. We simplified the thickness evolution of the PMMA layer at the short time scale with an exponential decay function, because we could not collect any XRR curves with Kiessig oscillations immediately after solution application. Moreover, we investigate thinner layers compared with Mouhamad *et al.* (2014 ▸), where the influence of the solution surface tension was introduced. This is not a critical parameter for our measurement because the solution is applied directly to the wafer spinning at a high speed and the initial drop is fragmented into multiple small drops after landing on the sample surface.

In summary, the spin-coating analysis using millisecond XRR makes it possible to resolve the detailed film parameters for two phases of PMMA film formation: the initial rapid thinning due to mass transport and a slower phase dominated by solvent evaporation. The film roughness initially decreases, stabilizes and then increases slightly due to porosity as the solvent evaporates. The study underscores the efficiency of combining *refnx* for fitting a few prototypical scans and *mlreflect* for fitting real time series with tens of thousands of qXRR curves for film parameter analysis during spin coating.

## 5. Conclusions

This study demonstrates the feasibility of fast millisecond XRR measurements on a static gold thin film sample as a reference and also applies it to an *in situ* PMMA spin-coating process. We show that qXRR can investigate surface and interface dynamics on a millisecond scale, which is an order of magnitude faster than previous applications of qXRR. The accuracy and precision of our experimental approach were substantiated by quantitative fitting of qXRR measurement results on a thin gold layer that agrees within error bars with much slower standard XRR scans. These findings underscore the utility of millisecond XRR as a powerful tool for probing and understanding rapid structural transformations in thin film processes.

Our findings not only highlight the power of millisecond XRR for real time investigations but also underscore its utility in monitoring complex dynamic processes at the nanoscale. It paves the way for more extensive applications of millisecond XRR in diverse areas of science and technology, including thin film deposition from vacuum and solution polymer processing, but also for investigating structural changes due to effects such as photostriction, diffusion and intercalation processes that occur on relevant time and length scales. When combined with on-the-fly NN analysis (Pithan *et al.*, 2023 ▸), it holds the potential for fast online analysis of nanoscale dynamics and potentially for process control as well. As we continue to refine and expand the capabilities of this methodology, we anticipate that it will play an instrumental role in advancing our understanding of dynamic surface phenomena and the development of novel materials and devices.

## Supplementary Material

Supporting figures and equations. DOI: 10.1107/S1600576724001171/jo5099sup1.pdf

Animation of the sample from the top. DOI: 10.1107/S1600576724001171/jo5099sup2.avi

Animation of the sample from a side. DOI: 10.1107/S1600576724001171/jo5099sup3.avi

## Figures and Tables

**Table 1:** Fit results for a standard 200 s-long XRR measurement, a rapid 1.4 ms qXRR measurement and an integrated 30 s-long qXRR measurement (uncertainities from the *refnx* fit are given)

XRR measurement	Thickness (Å)	Roughness (Å)	SLD (10^−6^ Å^−2^)
Standard XRR	291.9 ± 0.2	5.3 ± 0.1	120 ± 1
qXRR (1.4 ms)	291.7 ± 0.3	5.4 ± 0.1	118 ± 2
qXRR (30 s)	291.4 ± 0.2	5.4 ± 0.1	121 ± 1

**Table 2:** Overview of the results for the investigated PMMA solutions and spin-coated frequencies, the final thickness for each spin-coating experiment, and the delay between solution application and the first XRR spectra with fittable Kiessig fringes are given

Rotation speed (Hz)	qXRR acquisition (ms)	Concentration (g l^−1^)	Final thickness (Å)	Delay (ms)
30.3	8.25	2.5	134	2180
		5	225	2586
40	6.25	2.5	102	1590
		5	183	1648
66.6	3.75	2.5	81	1050
		5	142	1170
100	2.5	2.5	79	740
		5	130	850
175	1.4	2.5	60	445
		5	111	260
