# Supplementary material for: Millisecond X-ray reflectometry and neural network analysis: unveiling fast processes in spin coating
Source: J Appl Crystallogr. 2024 Mar 15;57(Pt 2):314–23. doi: 10.1107/S1600576724001171 (PMC11001405; doi:10.1107/S1600576724001171)
Supplement: Supplementary file 1 [file j-57-00314-sup1.pdf]

JOURNAL OF
APPLIED
CRYSTALLOGRAPHY

Volume 57 (2024)

Supporting information for article:

Millisecond X-ray reflectometry and neural network analysis: unveiling fast processes in spin-coating

David Schumi-Mareček, Florian Bertram, Petr Mikulík, Devanshu Varshney, Jiří Novák and Stefan Kowarik

Millisecond X-ray reflectometry and neural network analysis: Unveiling fast processes in spin-coating

1. Calculation of the footprint correction of the rotating sample

The sample is glued on the top of a wedge with either a steepness ω_{max} = 1° or ω_{max} = 2°. The sample has a square shape with the length of the edge L = 1 cm and was placed onto the wedge such that the sample sides are aligned with the beam when φ = 0°. We assume that the maximum angle of incidence is achieved when φ = 90°, for φ = 0° and φ = 180° the incident angle is 0° (beam parallel with the surface) and the beam is hidden behind the sample from φ = 180° to φ = 360° (second half of the rotation). From this, we can calculate an active sample size during the rotation.

$$l_{active} = \begin{cases} \dfrac{L}{\cos(90 - |\varphi|)}, & \varphi \geq 45 \\ \dfrac{L}{\cos(|\varphi|)}, & \varphi < 45 \end{cases} \tag{S1}$$

Where l_{active} is the active length of the sample and L is the length of the sample edge. The function is symmetric and therefore it is sufficient to calculate l_{active} just for φ = <0,90>. The footprint correction for the rotating squared sample can be calculated for each ω as:

$$F = \frac{b}{l_{active} \sin \omega} \tag{S2}$$

Where b is the beam size and it is applied for ω for which F is higher than 1. The relation between φ and ω is not linear and therefore we need to calculate φ from ω to keep the same order as the measured data. We can calculate φ as follows:

$$\varphi = \sin^{-1} \frac{\omega}{\omega_{max}} \tag{S3}$$

The complete derivation of the equation is given in the next section.

2. Reflectometry from the rotating sample

The beam reflected from the rotating sample doesn't move up and down on a line but creates a teardrop-curve shape when it goes from ω = 0 degree to ω_{max} and back to ω =0 degree (Fig. 1 (a) and two animations added to the SI).

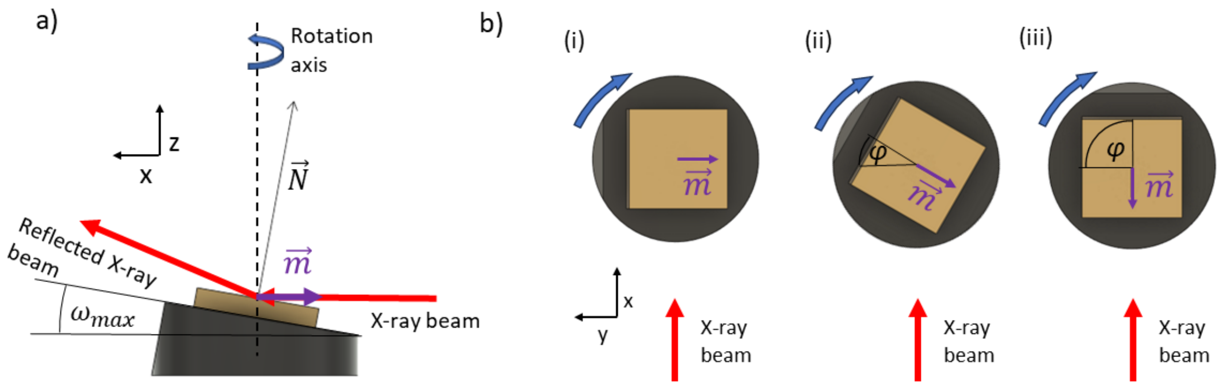

Figure 1

Figure S1 (a) Side view of the sample on the top of the wedge with an angle of $\omega_{\max}$. The rotation axis of the spin coater is perpendicular to the incoming X-ray beam. The $\vec{m}$ vector points towards the lower side of the wedge. (c) Top view with three examples of the sample position during the rotation from the top view. (i) The sample position where the incident angle is zero and the X-ray beam is not reflected as it goes parallel with the surface ($\vec{m}$ is perpendicular to the incoming X-ray), ω=0°. (ii) The position of the sample where φ = 30°. (iii) The maximum incident angle $\omega = \omega_{\max}$ of the sample is reached at φ = 90° as depicted in a side view in (a).

To understand the shape of the X-ray reflected beam on the detector we had to calculate the reflected beam from the rotating sample. The reflected vector can be calculated from the incoming beam and the surface normal vector using:

$$\vec{K}_{out} = \vec{K} - 2(\vec{K} \cdot \vec{n}\,)\, n \tag{S4}$$

Where incoming beam $\vec{K}$ is considered as (1,0,0) and $\vec{n}$ is the surface normal vector. The projection of the normal surface vector of the sample on the wedge with the steepness ω_{max} into the *xy* plane can be written as:

$$|m| = \sin \omega_{max} \tag{S5}$$

If the sample rotates the *z* part of the vector stays constant, but the *x* and *y* are changing with φ (Fig. 1). Therefore, the complete surface normal vector is defined as:

$$\vec{n} = \begin{matrix} -\sin\omega_{max} * \sin\varphi \;\; \hat{\imath} \\ -\sin\omega_{max} * \cos\varphi \;\; \hat{\jmath} \\ \cos\omega_{max} \;\; \hat{k} \end{matrix} \tag{S6}$$

With a usage of the reflectivity equation (4) and defined $\vec{K}$ and $\vec{n}$, we calculate,

$$\vec{K}_{out} = \begin{matrix} 1 - 2(\sin^2\omega_{max} * \sin 2\varphi)\ \hat{\imath} \\ -\sin^2 \omega_{max} * \cos^2 \varphi\ \hat{\jmath} \\ \sin 2\omega_{max} * \cos \varphi\ \hat{k} \end{matrix} \tag{S7}$$

For the beam shape on the detector, we must calculate the intersection of the reflected beam and the 2D detector plate (*yz*) at the correct sample-detector distance as follows:

$$\begin{aligned} y_d &= \tan \sin^{-1} K_{outy} * c \\ z_d &= \tan \sin^{-1} K_{outz} * c \end{aligned} \tag{S8}$$

where K_{outz} and K_{outy} are *z* and *y* coordinates of the beam outcoming vector $\vec{K}_{out}$ and *c* is the distance between the sample and the detector. In our experimental setup, the distance *c* was measured as 111 cm and in Figure S1 (b) we show the fit of the reflected beam on the top of the detector image created from the reflection of the rotating silicon sample with a thin Au layer on the wedge with $\omega_{max} = 1°$.

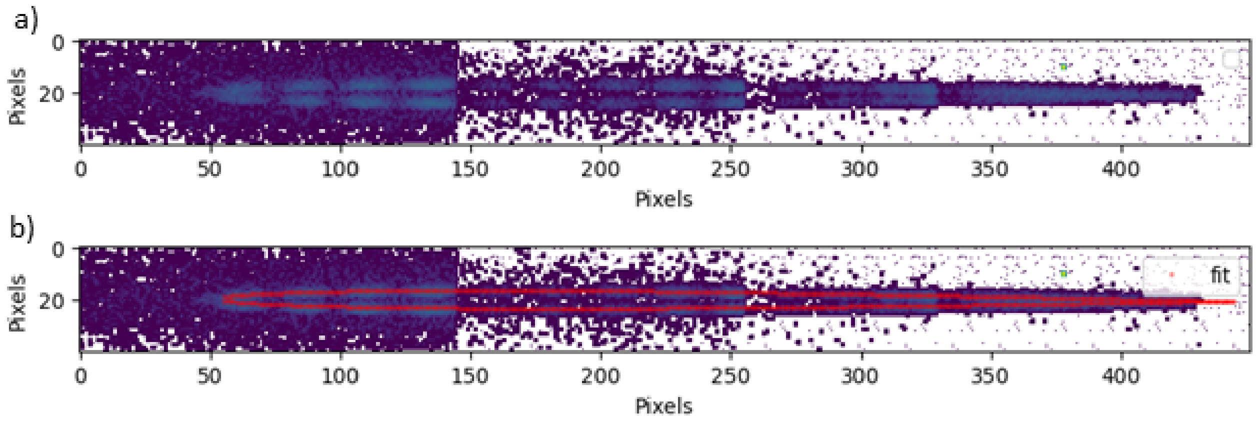

Figure S2 (a) The path of the specularly reflected beam on the 2D detector. (b)The fit of the specularly reflected beam according to equation (8).

Arising from the specific geometry of the presented qXRR experiment another normalization of the signal is needed, namely the velocity of the specular reflected beam over the detector. As explained above, the movement of the reflected beam is not linear in time, and the pixel exposure time is not constant. The speed can be calculated as $\frac{d\vec{K}_{out}}{d\varphi}$, which can be written as

$$\begin{aligned} \dot{K}_{outy} &= \sin^2 \omega_{max}\, 2 \cos 2\varphi \\ \dot{K}_{outz} &= \sin 2\omega_{max} \sin \varphi, \end{aligned} \tag{S9}$$

the partial derivatives in y and z direction. By incorporating the obtained result into equation S8, we can calculate the values of $\dot{y}_d$ and $\dot{z}_d$ which are utilized for the final normalization of the qXRR curve.

3. Absorbers

The shielding factor for each absorber plate was determined through one-minute-long measurements on a direct beam. We compared the obtained counts at each absorber set up and we calculated a shielding factor for the plates that we used for the normalization of the detector signal.

4. Fitting PMMA

One box model

After each spin coating run, we measured the PMMA layer by a “long” exposure measurement (30 seconds) (fig. S2). This measurement gave us a qXRR curve with a significantly lower noise background and can be used to fit the data with refnx and obtain the final thickness, roughness and SLD of the spin-coated layer. These parameters were critical for the data simulation for the NN training. For the experiment with PMMA solution with the concentration of 5 g/l and rotation of 30.3 Hz, the refnx fit (fig. 2) parameters were thickness 223.8 Å, roughness 5.6 Å and SLD 6.2 10^{-6}Å^{-2}. Moreover, we can even see the small oscillations created by the 100 nm thick thermal silicon dioxide layer.

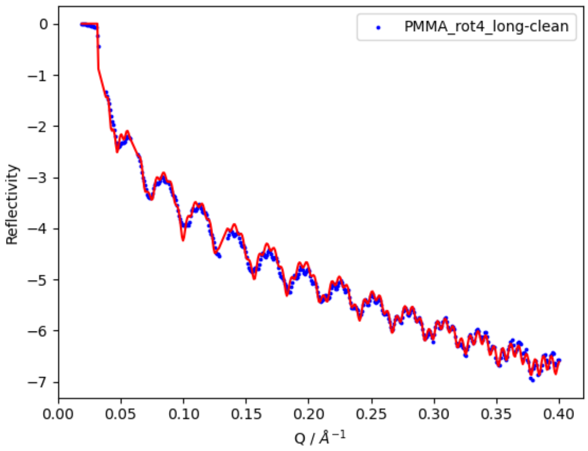

Figure S3 Long exposure (30 seconds) qXRR of PMMA (5 g/l solution) after the spin-coating at 30.3 Hz on a silicon wafer with silicon oxide layer with clear oscillations from PMMA and thick SiO2 layer. Fitted parameters (refnx) of the PMMA layer were as follows, thickness 223.8 Å, roughness 5.6 Å and SLD 6.2 10^{-6}Å^{-2}.

Three box model

For a few XRR curves we observed a shape hinting at a more complicated internal structure (Wu *et al.*, 1994). The solution with a concentration of 2.5 g/l (Fig. S4) and 1 g/l (Fig. S5) were spin-coated at the speed of 30.3 Hz. In these most extreme cases of our preparation conditions, we compared the two-box and the three-box models to show the accuracy of the fits. For the solution with the concentration of PMMA 2.5 g/l the χ^2 error for the two-box model was 4.3 and for the three-box model 3.4. For the concentration of 1 g/l the χ^2 error for the two-box model was 5.2 and for the three-box model 3.5. From the fit (fig.S4 and S5), we can see that the semifluid and interlayers are relatively thin and the SLD is lower than the SLD of the bulk PMMA. Compare this to the one-box model where the χ^2 error was 7.7 for the concentration of 2.5 g/l and 6.7 for the concentration of 1 g/l.

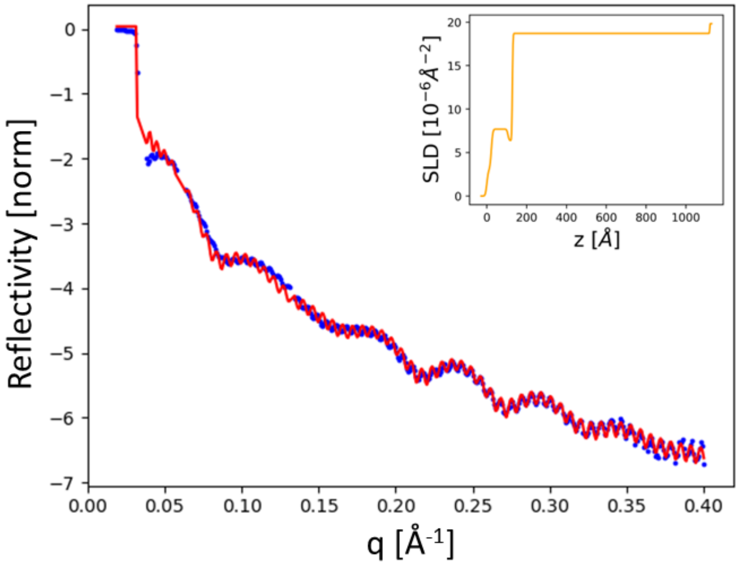

Figure S4 Three-box model fit of the spin-coated solution with 2.5 g/l of PMMA at 30.3 Hz. The parameters of the fit were as follows: Semifluid layer: Thickness = 22.2 Å, roughness = 6 Å and SLD = 3 10^{-6}Å^{-2}. Bulk: Thickness = 76 Å, roughness = 6.2 Å and SLD = 7.2 10-6/Å2. Interlayer: Thickness = 29.2 Å, roughness = 5 Å and SLD = 6.3 10^{-6}Å^{-2}. An SLD profile of the sample is inserted.

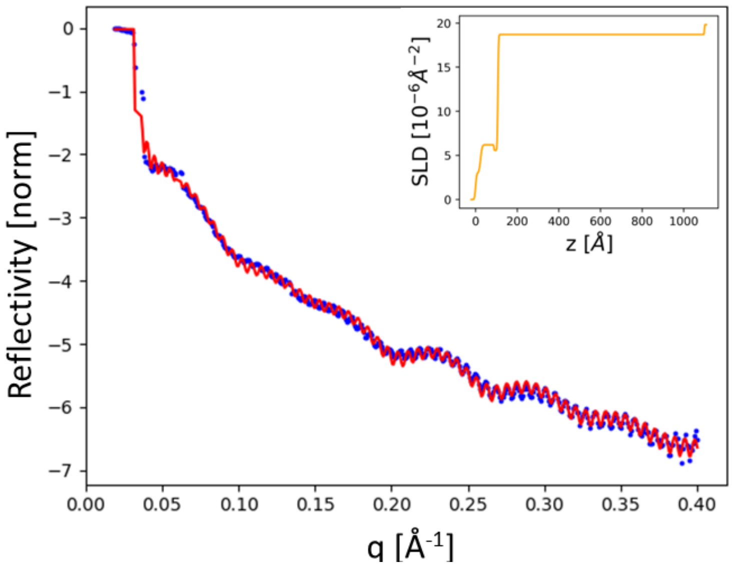

Figure S5 Three-box model fit of the spin-coated solution with 1 g/l of PMMA at 30.3 Hz. The parameters of the fit were as follows: Semifluid layer: Thickness = 23 Å, roughness = 5 Å and SLD = 2.8 10-6/Å2. Bulk: Thickness = 55.5 Å, roughness = 8 Å and SLD = 7 10-6/Å2. Interlayer: Thickness = 28.2 Å, roughness = 5 Å and SLD = 6 10-6/Å2. An SLD profile of the sample is inserted.

It is important to note that the more complicated model is always going to have a lower χ^2 error because of the possible overfitting. Moreover, the differences in the XRR curves shown in Fig. S4 and Fig. S5 could be caused by the inhomogeneity of the spin-coated layer. In the center of the sample, the PMMA layer can be significantly thicker than on the edges because the centrifugal force is weaker closer to the centre of rotation and stronger on the edges. For lower ω the beam is reflected from the all-sample length and the signal is averaged over the entire sample. However, for higher ω the beam footprint is smaller than the sample size and the beam hits only the central part of the sample where the PMMA layer can be thicker. This hypothesis was proven by the measurement of the samples at the X-ray source at the home laboratory, Where we measured that for the concentration of 2.5 g/l the layer thickness was 106 Å and 88 Å in the middle and on the side of the sample respectively. At a concentration of 1 g/l, the layer thickness was found to be 102 Å in the middle and 75 Å on the side of the sample.

5. Spincoating procedure:

We outline the step-by-step process of the spin coating measurements conducted in our experiments. Consistency was maintained by following the same sequence of steps for each experiment:

1. Test qXRR: Prior to the spin coating experiment, we initiated a test qXRR to set the appropriate time constants in the beam line macro. This step helped us to optimize the measurement parameters.
2. Data Collection from the silicon wafer: During the spin coating experiment, we collected data from each rotation, resulting in two XRR curves per rotation. At the initial stage of the spin coating process, we allowed the sample to spin without applying the PMMA solution. This allowed us to obtain data from the bare wafer surface (Fig. 3 (a)).
3. Application of PMMA Solution: After approximately 15 seconds, we applied the PMMA solution to the spinning sample. This caused an immediate change in the XRR spectra as the X-ray beam gets absorbed and scattered by the solution droplet on the sample surface (Fig. 3 (b)).
4. Observation of Kiessig Oscillations: After a few seconds (depending on the rotation speed, table 1, main text), the reflectivity edge reappeared, and we began to observe Kiessig oscillations from the PMMA layer in the XRR spectra (Fig. 3 (c)). However, it is important to note that due to the sensitivity of X-ray reflectivity to film structure, we were unable to detect Kiessig oscillations in regions where the layer exhibited high surface roughness.
5. Data Collection from PMMA: We continued to collect data for two minutes to observe changes in the PMMA layer over time(Fig. 3 (d)).
6. Long Exposure: At the end of the spin coating process, while keeping the sample rotating, we performed a long exposure qXRR (similar to Figure S3) to collect an XRR curve with a lower noise level for later fitting.

By following these steps consistently, we were able to perform the spin coating measurements and analyze the resulting XRR data.

6. All spin-coating runs

Here we show an examination of thickness evolution in all spin-coated thin films produced from a PMMA solution with a concentration of 5 g/l (Fig. S6) and 2.5 g/l (Fig. S7). The initial phase is characterized by an exponential decrease in thickness, leading to the establishment of a mechanically stable thickness. The temporal offset from t = 0 (solution application) is determined by the time required for the spin-coated layer to develop a smooth film with Kessigs oscillations. Subsequently, a noticeable thinning of the layer ensures, attributed to the evaporation of toluene. Significantly, the drying process is accelerated for thinner layers, underscoring the influence of the initial thickness of the PMMA layer on this phenomenon.

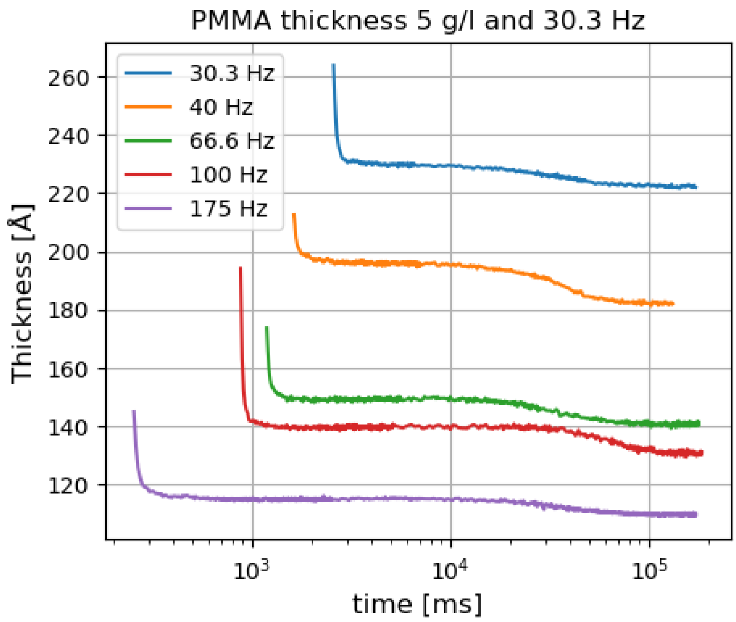

Figure S6 PMMA concentration 5 g/l. Depiction of the thickness evolution of spin-coated thin films fabricated from a 5 g/l PMMA solution. Initially, an exponential decay in thickness is evident, leading to the attainment of a mechanically stable thickness. Subsequently, thinning of the layer occurs as a result of toluene evaporation, with the drying process observed to be more rapid for thinner layers.

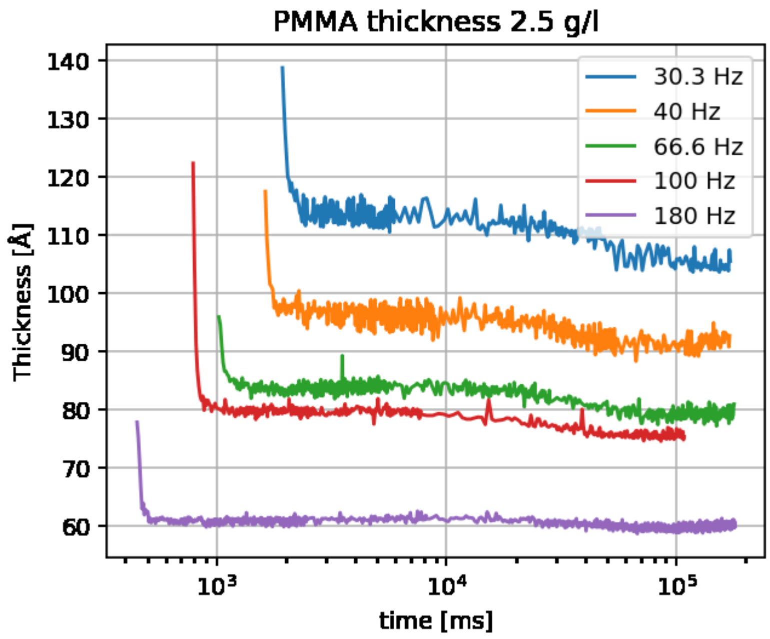

Figure S7 PMMA concentration 2.5 g/l. Depiction of the thickness evolution of spin-coated thin films fabricated from a 2.5 g/l PMMA solution. Initially, an exponential decay in thickness is evident, leading to the attainment of a mechanically stable thickness. Subsequently, thinning of the layer occurs as a result of toluene evaporation, with the drying process observed to be more rapid for thinner layers.

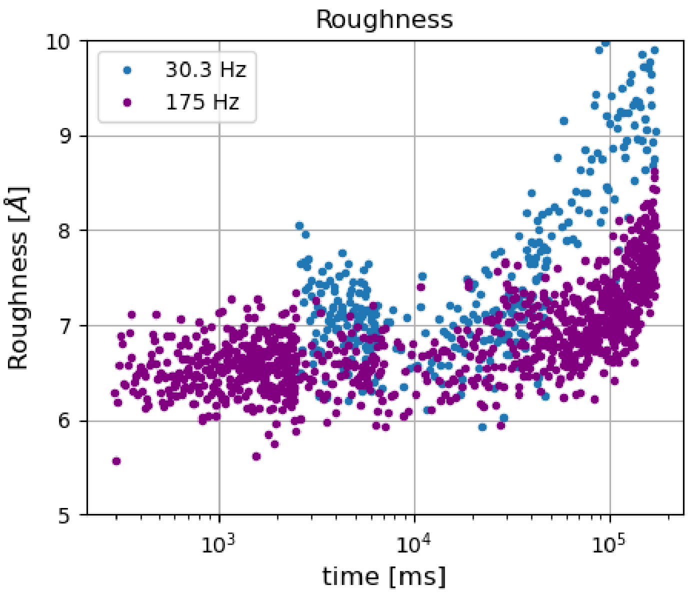

Figure S8 Comparison of a roughness evolution for two spin-coated films at 30.3 Hz (blue) and 175 Hz (purple). The roughness for the thicker film increases to higher values than for the thin layer.

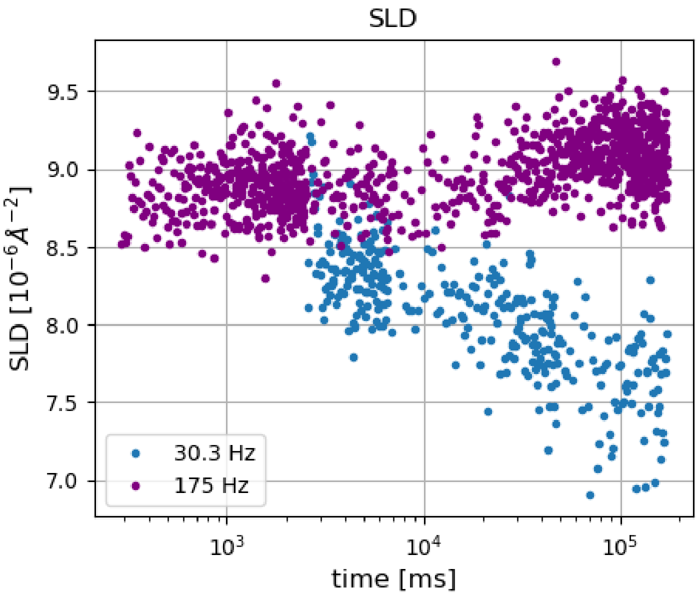

Figure S9 Comparison of an SLD evolution for two spin-coated films at 30.3 Hz (blue) and 175 Hz (purple). The SLD for the thicker film decreases as the toluene evaporates and pores are filled up with nitrogen. The thinner layer keeps the stable SLD during the measurement.

7. Individual fits of spin-coating experiments

Here we provide single-box model fits of XRR curves collected during the fast initial film thinning for all spincoating experiments. We also provide the plot of thickness evolution that was fitted with the

exponential decay same as shown in Fig. 5. In each figure, we always show 1st, 3th, 5 th, 7 th and 9 th fitted curves from the corresponding thickness evolution plot. The sampling speed depends on the rotation speed and varies from 8.25 millisecond to 1.4 millisecond for 30.3 Hz and 175 Hz respectively. We analysed only one curve per revolution.

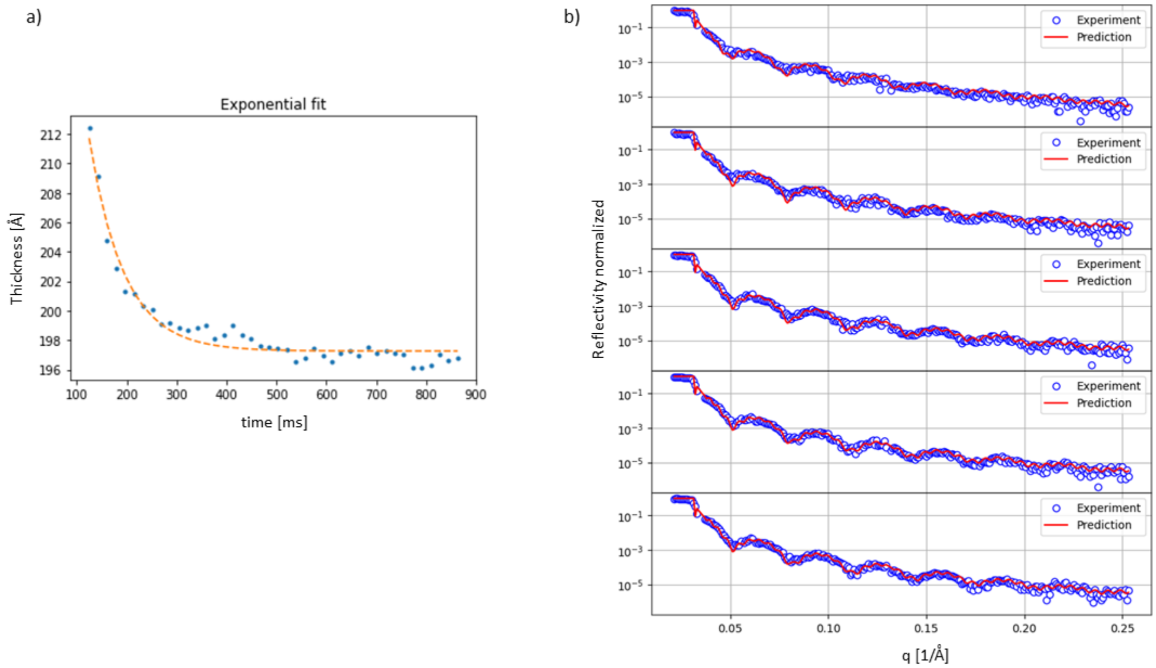

Figure S10 (a) Thickness evolution of PMMA layer at the short time scale fitted with an exponential decay function. (b) Fit of XRR data by NN for five representative XRR curves during the fast PMMA thinning phase (concentration 5 g/l and rotation of 40 Hz).

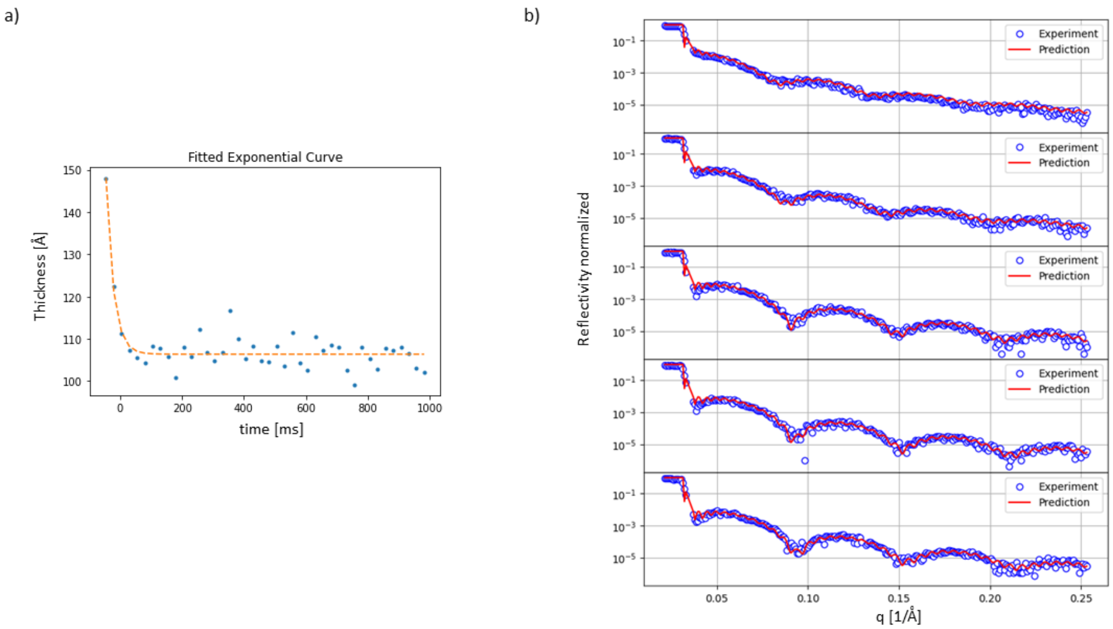

Figure S11 (a) Thickness evolution of PMMA layer at the short time scale fitted with an exponential decay function. (b) Fit of XRR data by NN for five representative XRR curves during the fast PMMA thinning phase (concentration 2.5 g/l and rotation of 40 Hz).

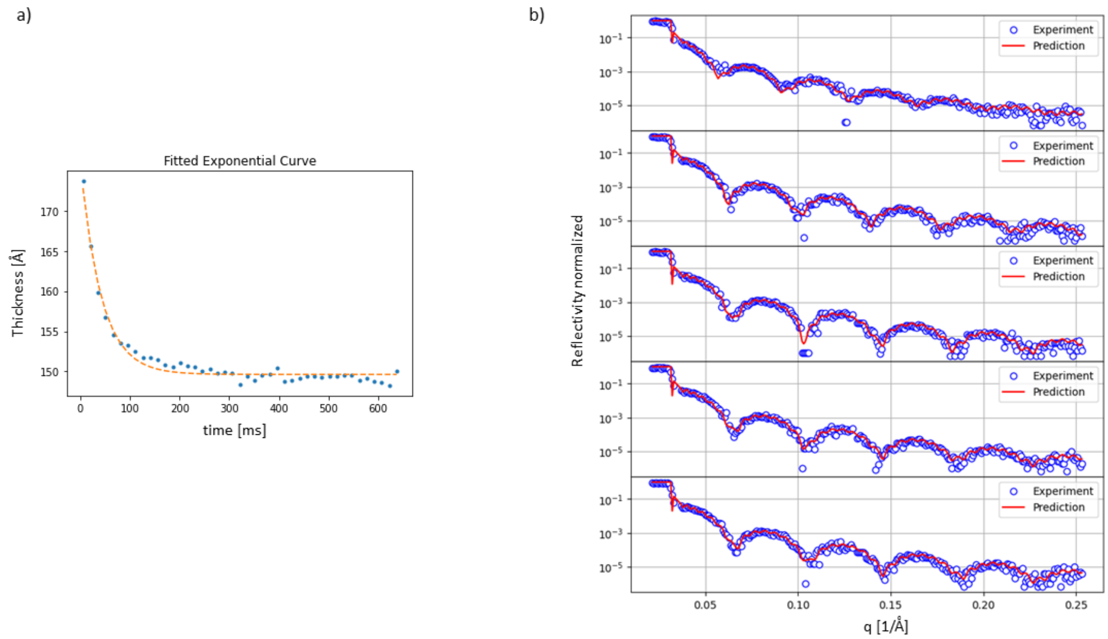

Figure S12 (a) Thickness evolution of PMMA layer at the short time scale fitted with an exponential decay function. (b) Fit of XRR data by NN for five representative XRR curves during the fast PMMA thinning phase (concentration 5 g/l and rotation of 67 Hz).

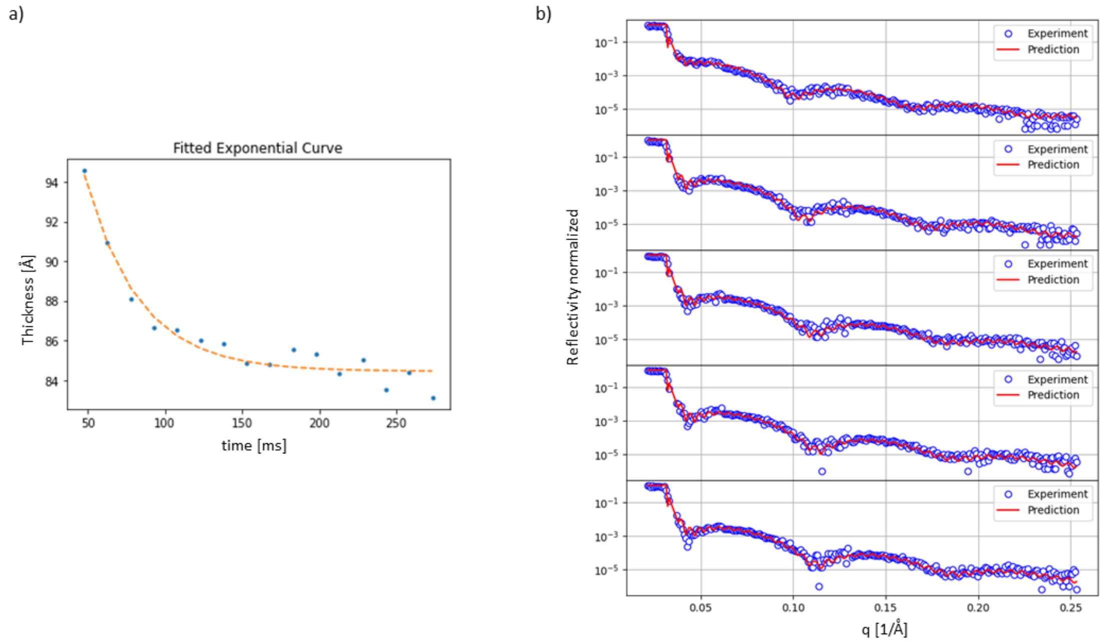

Figure S13 (a) Thickness evolution of PMMA layer at the short time scale fitted with an exponential decay function. (b) Fit of XRR data by NN for five representative XRR curves during the fast PMMA thinning phase (concentration 2.5 g/l and rotation of 67 Hz).

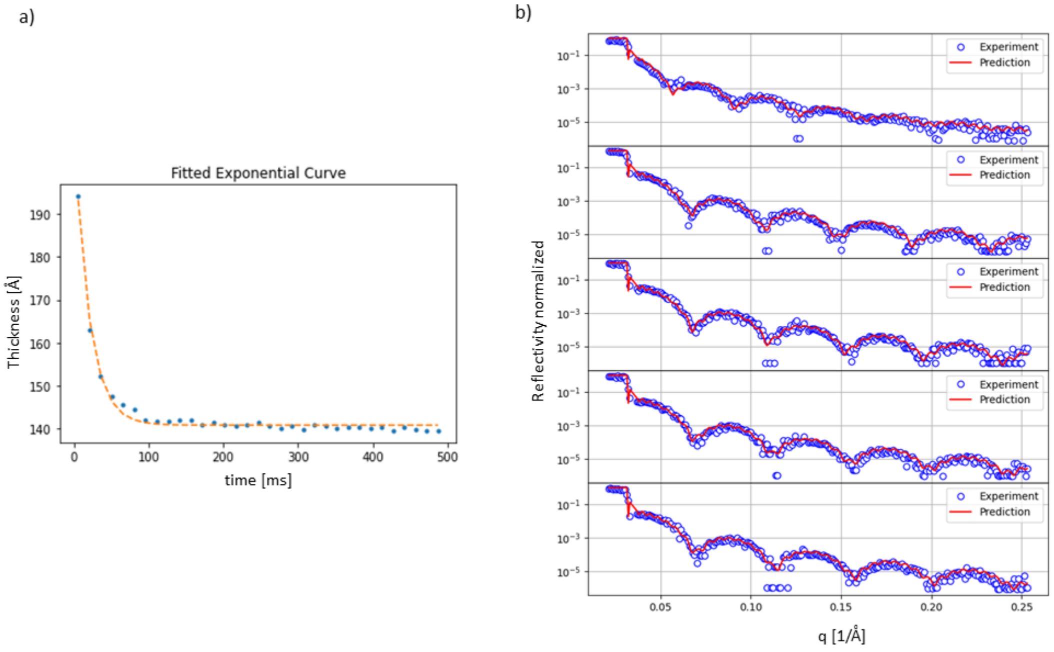

Figure S14 (a) Thickness evolution of PMMA layer at the short time scale fitted with an exponential decay function. (b) Fit of XRR data by NN for five representative XRR curves during the fast PMMA thinning phase (concentration 5 g/l and rotation of 100 Hz).

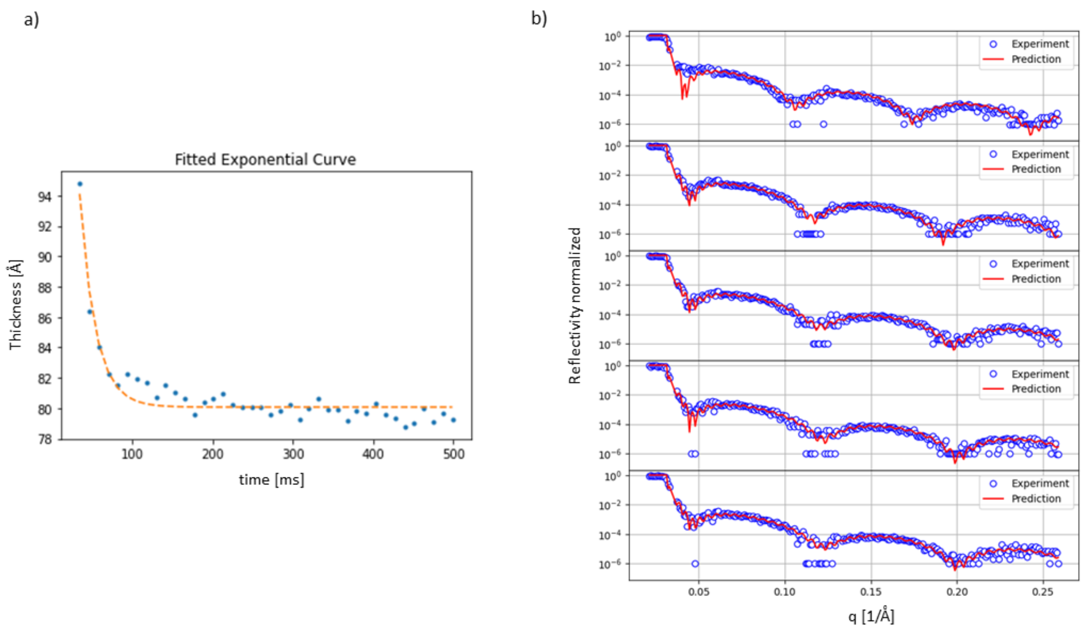

Figure S15 (a) Thickness evolution of PMMA layer at the short time scale fitted with an exponential decay function. (b) Fit of XRR data by NN for five representative XRR curves during the fast PMMA thinning phase (concentration 2.5 g/l and rotation of 100 Hz).

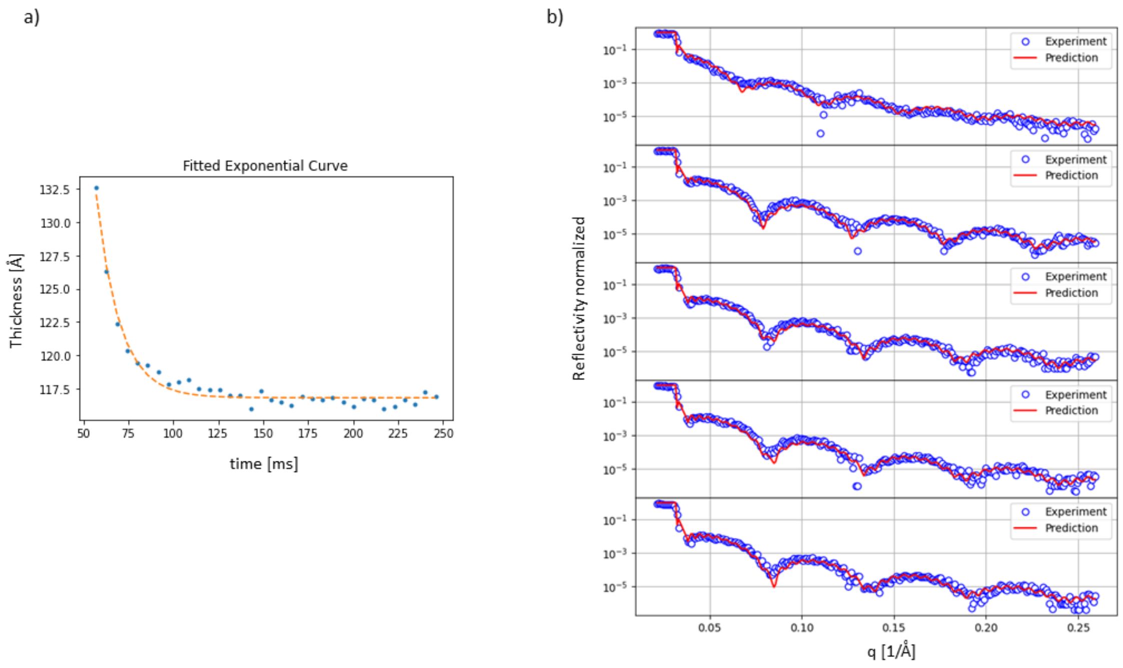

Figure S16 (a) Thickness evolution of PMMA layer at the short time scale fitted with an exponential decay function. (b) Fit of XRR data by NN for five representative XRR curves during the fast PMMA thinning phase (concentration 5 g/l and rotation of 175 Hz).

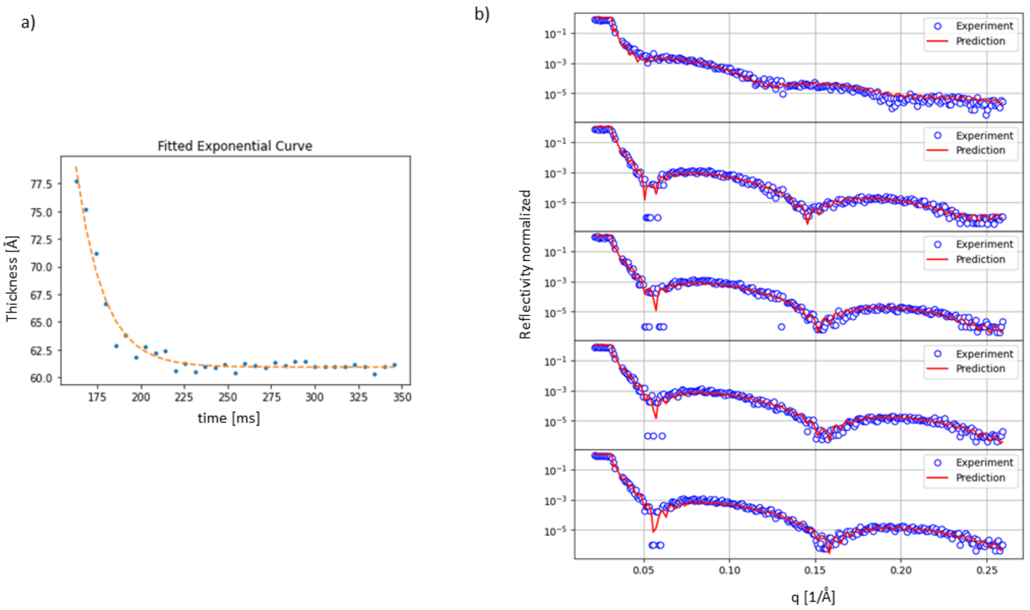

Figure S17 (a) Thickness evolution of PMMA layer at the short time scale fitted with an exponential decay function. (b) Fit of XRR data by NN for five representative XRR curves during the fast PMMA thinning phase (concentration 2.5 g/l and rotation of 175 Hz).

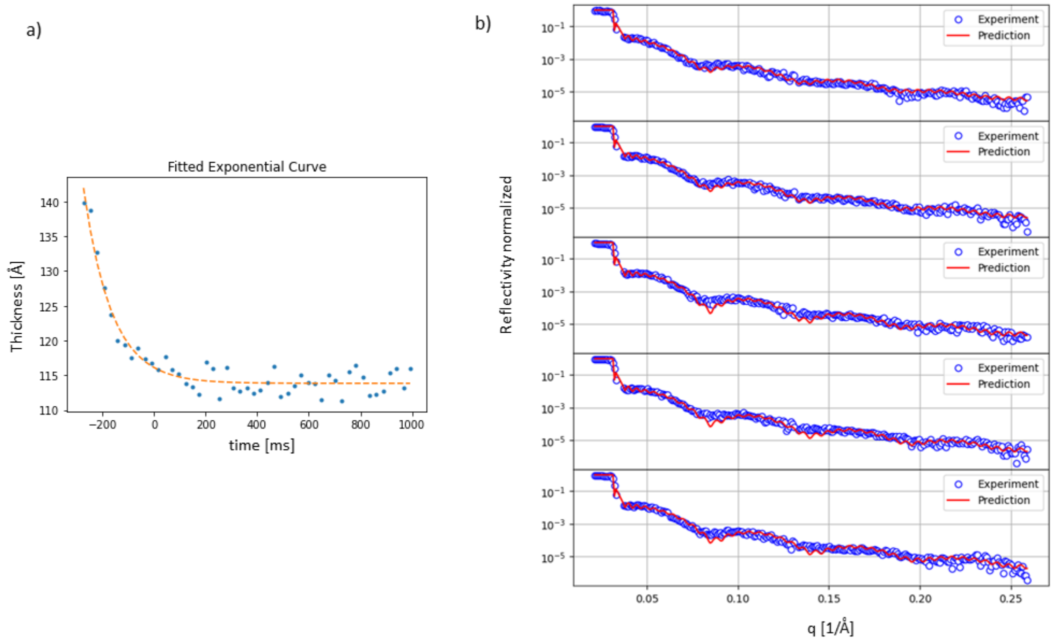

Figure S18 (a) Thickness evolution of PMMA layer at the short time scale fitted with an exponential decay function. (b) Fit of XRR data by NN for five representative XRR curves during the fast PMMA thinning phase (concentration 2.5 g/l and rotation of 33 Hz). This case is special, and fits have higher χ^2 error because the film was inhomogeneous and the film was thicker in the centre of the sample than on the edges. For more information see Fig. S4 & S5.

References:

Wu, W. -l, Orts, W. J., Van Zanten, J. H. & Fanconi, B. M. (1994). *J Polym Sci B Polym Phys* **32**, 2475–2480.
